# Roles of HTLV-1 basic Zip Factor (HBZ) in Viral Chronicity and Leukemic Transformation. Potential New Therapeutic Approaches to Prevent and Treat HTLV-1-Related Diseases

**DOI:** 10.3390/v7122952

**Published:** 2015-12-09

**Authors:** Jean-Michel Mesnard, Benoit Barbeau, Raymond Césaire, Jean-Marie Péloponèse

**Affiliations:** 1 CPBS, CNRS FRE3689, Université Montpellier, 34293 Montpellier, France; jean-michel.mesnard@cpbs.cnrs.fr; 2 Département des Sciences Biologiques, and Centre de Recherche BioMed Université du Québec à Montréal, Montréal, QC H2X 3X8, Canada; barbeau.benoit@uqam.ca; 3 Laboratoire de Virologie-EA4537, Centre Hospitalier et Universitaire de Martinique, Fort de France, Martinique; Raymond.Cesaire@chu-fortdefrance.fr

**Keywords:** human T-cell leukemia virus type 1, adult T-cell leukemia, HTLV-1 bZip Factor, Valproate

## Abstract

More than thirty years have passed since human T-cell leukemia virus type 1 (HTLV-1) was described as the first retrovirus to be the causative agent of a human cancer, adult T-cell leukemia (ATL), but the precise mechanism behind HTLV-1 pathogenesis still remains elusive. For more than two decades, the transforming ability of HTLV-1 has been exclusively associated to the viral transactivator Tax. Thirteen year ago, we first reported that the minus strand of HTLV-1 encoded for a basic Zip factor factor (HBZ), and since then several teams have underscored the importance of this antisense viral protein for the maintenance of a chronic infection and the proliferation of infected cells. More recently, we as well as others have demonstrated that HBZ has the potential to transform cells both *in vitro* and *in vivo*. In this review, we focus on the latest progress in our understanding of HBZ functions in chronicity and cellular transformation. We will discuss the involvement of this paradigm shift of HTLV-1 research on new therapeutic approaches to treat HTLV-1-related human diseases.

## 1. Introduction

Thirty years ago, human T-cell leukemia virus type 1 (HTLV-1) was the first human retrovirus to be identified and is now known as the causative agent of a very aggressive form of leukemia termed adult T-cell leukemia (ATL). It was isolated in the early 1980s, first in the United States [1] and then in Japan [2,3]. Currently, HTLV-1 infects approximately 15 million individuals worldwide [4]. HTLV-1 is the etiological agent of both ATL and a slowly progressive neurologic disorder called HTLV-1-associated myelopathy/tropical spastic paraparesis (HAM/TSP). [5,6]. The role of HTLV-1 in HAM/TSP will not be discussed here. Below, we summarize and update insights relevant to human leukemogenesis induced by HTLV-1.

## 2. HTLV-1 Infectivity and Spread *in vivo*

**Figure 1:**
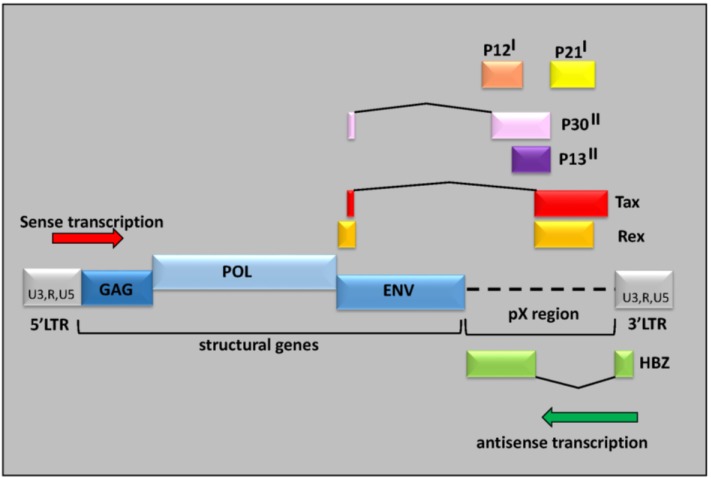
Structure of the HTLV-1 provirus: The human T-cell leukemia virus type 1 (HTLV-1) genome encodes for three structural proteins, Gag, Pol, and Env, and complex regulatory proteins such as Tax, which not only activates viral replication, but also induces the expression of several cellular genes. The *in vivo* expression of these viral proteins is suppressed by cytotoxic T lymphocyte (CTL) activity. HTLV-1 basic Zip factor (HBZ), produced by a minus-strand mRNA, likely plays a role in viral replication and T-cell proliferation as it is steadily expressed in most HTLV-1-infected cells and primary adult T-cell leukemia (ATL) cells, whereas Tax is not.

Like any animal retrovirus, the HTLV-1 proviral genome encodes for the structural genes, *gag*, *pol* and *env*, and is bordered by two long terminal repeat sequences (LTR) [7]. The 5′ LTR serves as the main promoter for viral transcription. HTLV-1 is defined as a complex retrovirus because its genome also contains a region termed the pX region, which it located between the *env* gene and the 3′-LTR and contains genes encoding regulatory viral factors, Tax, Rex, p12^I^, p13^II^, p30^II^ and p21^I^. Furthermore, the minus strand of pX has been found to produce an antisense transcript, encoding HBZ [8,9,10,11] (Figure 1).

*In vitro*, HTLV-1 can infect a large variety of cells, including T- and B-cells, fibroblasts, macrophages, and dendritic cells [12]. These observations indicate that the receptor is common and expressed on a large number of cells. Studies show that glucose transporter 1 (Glut-1), heparan sulfate proteoglycans (HSPGs), and neuropilin-1 (NRP-1) are three proteins involved in the mechanism of HTLV-1 entry [13,14,15]. The current view on the entry event of HTLV-1 suggests that the virus first interacts with HSPG and then forms complexes with NRP-1 followed by an association with Glut-1 at the cell surface before final membrane fusion and entry into the cell. However, how these factors cooperate with each other requires further study. It is interesting to note that, despite the ubiquitous distribution of these membrane proteins, *in vivo*, the HTLV-1 provirus is mainly detected in CD4+ and in CD8+ T-cells [16]. There is nonetheless evidence that cellular receptors play an important role in determining the cellular tropism of HTLV-1 [17]. Thus, the differential outcome of HTLV-1 on CD4+ and CD8+ cell proliferation may be more important in dictating the apparent specificity for CD4+ cells than is receptor-binding and differences related to cellular entry [18].

*In vivo*, HTLV-1 is primarily transmitted by cell-to-cell contact, and not by cell-free virions [19]. Upon contact with an uninfected cell, HTLV-1-infected cells will transiently express high levels of Tax and intercellular adhesion molecule-1 (ICAM-1) to form a virological synapse [20] or a viral biofilm [21]. Enveloped viral particles can transfer through this synapse, thus propagating infection [22]. Recently, it has been reported that HTLV-1 cell-to-cell transmission is ten thousand times more efficient than cell-free infection, while, in comparison, similar experiments have shown that for human immunodeficiency virus type 1 (HIV-1) infection, this difference is only twofold [23]. However, the importance of *in vivo* cell-to-cell spread is tempered by findings that the administration of reverse transcriptase inhibitors (RTI) to HTLV-1-infected patients with HAM/TSP does not markedly influence the provirus load [24], and that RTI treatment immediately after HTLV-1 infection *in vivo* does not change subsequent proviral load. Thus, viral replication itself does not appear to be critical for the maintenance of persistent infection; rather, the proliferation of HTLV-1-infected cells seems to determine viral burden at the carrier state. In this regard, the viral strategy to increase the number of infected cells by promoting cellular proliferation is meaningful. Indeed, a long-standing observation is that HTLV-1 induces clonal proliferation of infected cells *in vivo* [18,25,26].

## 3. HTLV-1, Chronicity and Host Immune Response

In order to induce chronic infection, viruses need to establish an equilibrium between viral virulence and the host immunity [27]. Accordingly, human retroviruses, such as HTLV-1, have evolved several strategies to control the host immune system and temper viral replication, one of which is to directly deregulate the major histocompatibility complex (MHC) [27]. The function of MHC molecules is to bind peptide fragments derived from pathogens and display them on the cell surface for recognition by the appropriate T-cells. The consequences are often deleterious to the pathogen—virus-infected cells are killed and B-cells are activated to produce antibodies that eliminate or neutralize extracellular pathogens. Thus, there is a strong selective pressure in favor of any virus that has evolved mechanism allowing them to escape presentation of its antigens by MHC molecules. In its pX region, HTLV-1 encodes an accessory protein, p12 that interacts with MHC class I heavy chains, and leads to its degradation by the proteasome [28]. In HTLV-1-infected host, chronically activated cytotoxic T lymphocyte (CTL) response [29,30,31] and high titer of anti-HTLV-1 antibodies, mostly directed against the Tax protein [32,33,34], strongly support the idea that Tax is the main immunogenic target. Indeed, *in vivo* depletion of Tax-expressing CD4+ T-cells leads to moderate HTLV-1 replication [35]. CD8+ CTLs are in part responsible for this phenomenon because their depletion enhances Tax expression *in vivo* [35]. Furthermore, when a histone deacetylase inhibitor, valproate, was used to reactivate *tax* transcription in HTLV-infected host, their proviral load became reduced [36,37,38]. A similar observation of valproate-induced reduction of Simian T-Cell Leukemia Virus (STLV) proviral load has been also reported in a simian model [39]. Thus host’s CTL response targets Tax-expressing cells, thereby reducing the number of infected cells *in vivo*. In fact, the HTLV-1 proviral load appears to be maintained, when an equilibrium is established with the immune response, allowing the maintenance and the proliferation of HTLV-1-infected cells [40]. While Tax is frequently targeted by CTL in HTLV-1 infection [32,33,34], the frequency of HBZ-specific CTL is low and could only be detected in 25%–40% of infected individuals [41,42]. However, in a systematic study, MacNamara *et al.* [42] showed that protective alleles A*0201 and C*0801 bound HBZ-derived peptides with significantly higher affinity in comparison to alleles which were associated with disease progression (B*5401). However, further analyses demonstrated that asymptomatic carriers ACs had human leukocyte antigen (HLA) alleles which bound HBZ peptides significantly more strongly than patients with HAM/TSP, and that this difference in binding was not simply attributable to A*0201, C*0801, and B*5401 [41,42].

In order to escape the host immune response, a proportion of cells that express Tax must subsequently shut down its expression. Recently, various molecular mechanisms accounting for suppression of Tax expression have been suggested, implicating viral—Rex [43], the pX protein p30 II [44] and HBZ [8]—and cellular proteins—histone deacetylases [45] and GLI-2/THP [46]. In each study, these data only indicate a partial rather than a complete shutdown of proviral transcription. Importantly, the extent of suppression of viral expression in natural HTLV-1 infection is not yet known. However, even partial suppression should provide significant survival advantage to an HTLV-1-infected cell since these cells might be less prone to elimination by the immune system, which would be particularly dependent on CTL activity. Furthermore, impairment of CTL surveillance may similarly allow HTLV-1-transformed leukemic cells to survive and proliferate [47,48].

## 4. Multifaceted Processes in the Transformation of Infected Cells

Over time, a subset (2%–6%) of HTLV-1-infected individuals will develop ATL [49]. One of the current models is that the Tax oncoprotein confers survival and proliferative properties to infected cells (Figure 1) [50,51,52]. Tax is post-translationally modified by phosphorylation, ubiquitination, and acetylation [53,54,55,56,57,58,59]. These post-translational modifications have been shown to be important for Tax function [54,58]. Expression of Tax alone has been postulated to be sufficient for immortalization, but not transformation, of human T-cells [60,61]. The *in vivo* transforming capacity of Tax has been extensively investigated using transgenic mouse models; results suggest that Tax expression can solely drive *in vivo* tumor formation [62,63,64]. However, frequent appearance of type 2 defective proviruses (*i.e.*, lacking the 5′ LTR and the Tax gene) in ATL cells lead into questioning these current models [65,66,67]. Investigation of the mechanisms underlying the generation of these defective proviruses by Miyazaki *et al.* [67] showed that 41% of type 2 defective proviruses lacking 5′ LTR were formed before proviral integration. Since Tax expression alone is not enough to transform primary human cells *in vitro* [60,61,68], it is likely that, similar to human papillomavirus E6 and E7, which cooperate for the development of tumors [69], tax functions cooperatively with other HTLV-1-encoded genes in HTLV-1 to induce human leukemogenesis [63]. Further studies are required to better clarify the roles of the Tax in ATL onset.

## 5. HTLV-1 Antisense HBZ Transcripts and Viral Pathogenesis

While HTLV-1 plus strand (sense) contains transcripts driven from the 5′ LTR, the 3′ LTR produces an antisense transcript, which encodes a protein called HTLV-1 basic Zip factor , HBZ (Figure 1) [7]. The promoter for the *hbz* gene is contained in the U5 sequence of the 3′ LTR [7]. Analyses showed that HTLV-1 LTR possess a bidirectional transcriptional activity. Interestingly, Sp1 sites within this region are critical for the control of bidirectional and HBZ transcription [70,71]. While tax transcripts are detected in few transformed ATL cells, *hbz* mRNA is present in all ATL cells [72,73,74].

Through its basic Zip (bZIP) domain, the HBZ protein has been described to interact with transcription factors the cAMP-response element binding proteins (CREB, CREB-2), the cAMP-responsive element modulator (CREM-Ia) the activating transcription factor ATF-1 [75], c-Jun [76,77], JunB [78], and JunD [79], and was originally reported to suppress Tax-mediated viral transcription [8]. Furthermore, HBZ selectively inhibits the classical nuclear factor-kappa B (NF-κB) pathway by inhibiting DNA binding of p65 and promoting its degradation [80]. On the other hand, Tax has been shown to rather activate both classical and alternative NF-κB pathways [81]. Since the two pathways differentially control the expression of genes with anti-apoptotic functions in lymphoma cell lines [82], preferential activation of the alternative pathway by Tax and HBZ might be implicated in the proliferation of ATL cells. Interestingly, a previous study had suggested that the *hbz* mRNA itself is also important for the induction of proliferation of HTLV-1-infected cells [9]. Moreover, it has been shown that HTLV-1 molecular clones harboring a mutation in the leucine zipper domain of HBZ exhibit reduced proviral load compared to wild type virus when inoculated into rabbits [83]. Furthermore, HBZ has been reported to increase the activity of human telomerase reverse transcriptase (hTERT) [84]. Collectively, all of the available data support that HBZ protein and RNA play important roles in promoting viral replication and cellular proliferation [85].

Genetic instability in infected cells might allow them to escape strong CTL response, and further protect them from clonal dominance [86]. On the other hand, HTLV-1-triggered alterations in cellular gene expression have been proposed to amount to a mutator phenotype that promotes leukemogenesis [87]. Thus far, Tax has been recognized as the main source of HTLV-1-associated genetic instability. In a recent study, Vernin *et al.* [88] showed that HBZ promotes onco-miR expression as well as DNA-strand breaks by downregulating the expression of OBFC2A protein via posttranscriptional activation of miR17 and miR21. OBFC2A intervenes with ATM signaling and subsequently activates DNA repair and cell-cycle checkpoints [88]. In their study, Vernin *et al.* further suggested that preleukemic phenotypes of HTLV-1-positive CD4+ T cells is portrayed by an oncogenic miRNA profile that is promoted by HBZ [88].

The transforming capacity of HBZ has been clearly demonstrated *in vitro* and *in vivo* using HBZ transgenic (Tg) mice [89,90]*.* Using the 5′ LTR deleted K30_4089_ molecular clone [10], Gazon *et al.* have demonstrated that, in murine cells, HBZ expression on its own drives cellular proliferation and colony formation in soft agar [90]. Furthermore, *in vivo* studies showed that CD4+ Foxp3-positive T-cells from HBZ-Tg mice had similar effector/memory [89] and regulatory phenotypes to infected CD4+ T cells from ATL patients or HTLV-1-infected carriers [91,92] In this study by Satou *et al.*, transgenic mice expressing Tax under the same promoter as the HBZ-Tg mice did not display any changes in their Treg phenotype [89]. These data suggest that HBZ, rather than Tax, is responsible for conferring a specific phenotype to HTLV-1-infected cells and ATL cells.

**Figure 2:**
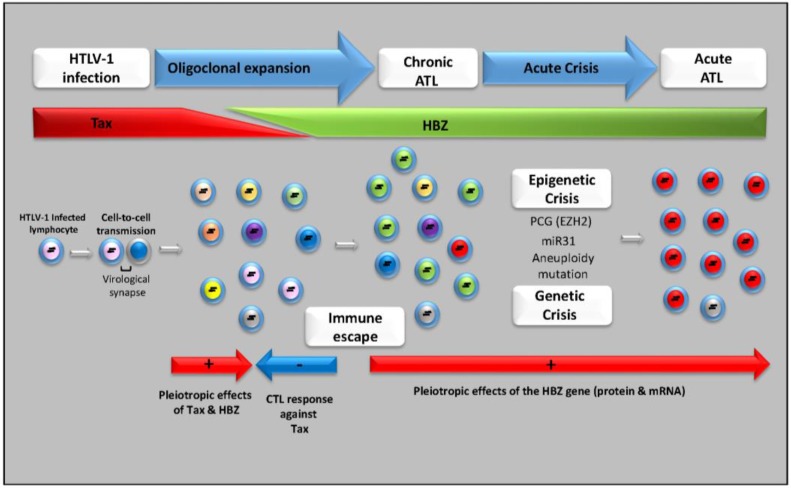
Model for ATL development. HTLV-1 is transmitted in a cell-to-cell fashion via a virological synapse. After infection, HTLV-1 promotes clonal proliferation of infected cells by pleiotropic actions of Tax and other viral proteins. Tax is considered crucial for the oligoclonal maintenance and expansion of HTLV-1-infected cells during the early phase but is only transiently expressed by HTLV-1-infected cells. Proliferation of HTLV-1-infected cells is controlled by cytotoxic T-cells *in vivo*. Thereafter, continuing expression of HBZ is followed by genetic/epigenetic loss of function of tumor suppressor genes and modulation of micro RNA levels. After a long latent period, ATL develops in about 5% of asymptomatic carriers. Diverse genetic abnormalities are acquired during the progression to ATL from an indolent to an aggressive disease form.

The interplay between Tax and HBZ in T-cell transformation might then be explained by the fact that the former would be needed to initiate transformation while the latter would be required to maintain the transformed phenotype of ATL cells at a time point when Tax expression is extinguished. However, when taking into consideration the accumulating evidence on HBZ functions, the notion that Tax only can initiate cellular transformation via its ability to induce genetic instability may require some revision. Indeed, it has been clearly established that HBZ is responsible for the specific phenotype, function and proliferation of HTLV-1-infected CD4+ T-cells and ATL cells, and that, in addition to Tax, HBZ plays an important role in the oncogenic activity of HTLV-1 (Figure 2). Furthermore, the long latent period observed by Satou *et al.* before the onset of T-cell lymphomas in HBZ-Tg mice suggests that additional epigenetic alterations in CD4+ T-cells are necessary for the development of T-cell lymphomas in HBZ-Tg mice as well as for ATL [89]. In conclusion, functions recently attributed to HBZ provide novel insights into the interaction between HTLV and its host and may be exploited to treat and prevent HTLV-1-induced diseases.

## 6. Therapeutic Approaches for ATL

ATL is an incurable and poorly treatable disease. Despite advances in both chemotherapy and supportive care, median survival time of patients remains less than one year [93]. As pointed out by Yamada and Tomonaga [94], an important amount of knowledge in molecular biology and oncogenesis of ATL has accumulated but has not yet been translated into improved prognosis of affected patients [94]. In fact, it has been reported that the prognosis of indolent subtypes, chronic and smoldering ATL, was 4.1 years, which is poorer than previously thought [95]. Therapeutic approaches using interferon-α combined with zidovudine have nonetheless been reported to be highly effective treatments for indolent ATL and, as they have been extensively reviewed, will not be further detailed here [96,97,98].

### 6.1. Chemotherapy

In Europe, US and Brazil, the recommended treatment of patients with acute or lymphoma-type ATL is based on the use of combined chemotherapy, as a first line therapy: cyclophosphamide, adriamycin, vincristine, and prednisolone (CHOP). 46.5% of patients treated with CHOP exhibit partial remission (PR), while only ~20% are achieving complete remission (CR) [99]. Intensification of CHOP with etoposide, vindesine, ranimustine, and mitoxantrone resulted in CR for 35.8% of ATL patients [100]. However, the median survival was only 8–8.5 months in these studies with predicted survivals of 13% after three years. In Japan, the first line of combined chemotherapy against ATL consists of VCAP-AMP-VECP) (i.e; vincristine, cyclophosphamide, doxorubicin, and prednisone (VCAP), doxorubicin, ranimustine, and prednisone (AMP), and vindesine, etoposide, carboplatin, and prednisone (VECP)) [101,102,103]. It has in fact been shown that the CR rate was better with VCAP-AMP-VECP (40%) than biweekly CHOP (~20%) and that a three-year survival rate of patients treated with VCAP-AMP-VECP therapy improved by 24% (*versus* 13% with CHOP treatment) [104]. The two main obstacles to combined chemotherapy are (1) the inherent drug resistance to chemotherapeutic agents observed in ATL cells [49] and (2) the profoundly weakened and immunodeficient state of ATL patients. Overall, ATL survival with various chemotherapy regimens is poor, with survival ranging between 5.5 and 13 months in several cohorts of patients, and more predominantly in patients with acute leukemia or lymphoma. At the moment, this approach does not represent a prospect for a cure.

### 6.2. Molecular Targeted Therapy

As an alternative to chemotherapy, a number of studies have addressed the potential use of nucleoside analogues for the treatment of ATL. The purine analog 2′-deoxycoformycin (DCF) that inhibits adenosine deaminase has been studied. In a phase II study using DCF, two CR (8%) and one PR (4%) cases were reported among 25 patients with ATL [105]. Unfortunately, these response rates are significantly lower than those with CHOP-based chemotherapy. Using DCF in conjunction with CHOP, 52% of ATL patients achieved CR, but the median survival of patients was only 7.4 months [106]. Interestingly, another study reported that a patient presenting resistant acute ATL had an improved and lasting partial response when treated with another adenosine analog, 2′-chlorodeoxyadenosine (cladribine) [107]. However the follow-up phase II study showed very limited benefit with this compound. It is nonetheless important to note that all patients under treatment showed resistant ATL prior to treatment in this study and therefore represented a poor prognosis group [108].

Parallel approaches, using analogs or inhibitors of topoisomerase, such as irinotecan hydrochloride (CPT-11) [109], a *bis* (2,6-dioxopiperazine) analog (MST-16) [110], Menogaril [111] or all-trans-retinoic acid (ATRA) [112,113], an analog of vitamin A have been similarly tested. The most promising results were obtained with the MST-16 treatment. In a cohort constituted of 21 acute-type ATL patients, treatment resulted in one CR and five PR. Among eight lymphoma-type ATL patients, two PR cases were identified, while out of two chronic-type ATL patients, one was diagnosed as being in CR while the other, as being in PR. Remissions were achieved within 23 days and lasted over two months (median, 68 days) [110]. These results do not clearly represent an improvement over conventional chemotherapy and further studies with MST-16 are needed.

Gene expression governed by epigenetic changes is crucial for the pathogenesis of cancer. Histone deacetylases are enzymes that are involved in the remodeling of chromatin and play a key role in the epigenetic regulation of gene expression. The use of histone deacetylase inhibitors (HDACi) to treat ATL has recently attracted attention. The HDACi LBH589 (panobinostat) exhibited significant anti-ATL activity by activating a novel RAIDD-caspase-2 pathway in mice and by modulating the expression of Tax and CCR4 [114]. However, a phase II study using panobinostat for cutaneous T-Cell Lymphoma (CTCL) and indolent ATL patients had to be terminated because of severe side effects and appearance of ulcers in patients with ATL [115].

Depsipeptide (HFR901228), another HDACi induces apoptosis in all tested HTLV-1-infected cell lines and in primary cells from patients with acute ATL, through a reduction of NF-κB and AP-1 transactivation activity, and downregulation of B-cell lymphoma-extra large (Bcl-xL) and cyclin D2 expression. Partial inhibition of tumor growth following transplantation of HTLV-1-infected cells, was seen in a severe combined immunodeficiency (SCID) mouse model [116]. Further studies are needed to evaluate the efficacy of HFR901228.

Sodium valproate (VPA) is another HDCAi under investigation, which is widely prescribed for the treatment of epilepsy, bipolar mood disorders, and migraine, and which shows HDACi activity among several other potential antitumor properties [117]. VPA is also being investigated for its inclusion in maintenance therapy after chemotherapy [118]. More importantly, dramatic clearance of both lymphoma and leukemic cells has been demonstrated in Bovine Leukemia Virus-induced B-cell malignancy in sheep upon treatment [37,119]. In a recent study, Belrose *et al.* [38] analyzed the impact of VPA treatment on the expression profile of Tax and HBZ in freshly cultured cells from HTLV-1-infected patients. It was then proposed that VPA relieved the epigenetic control over Tax expression, thereby exposing latently HTLV-1-infected cells to the immune response. Indeed, in the presence of VPA, Tax expression kinetics were profoundly modified, with Tax mRNA levels increasing constantly over time, suggesting dysregulation of the processes responsible for the control of its expression in lymphocytes from HAM/TSP patients, but not from asymptomatic carriers. One interesting finding from the Belrose *et al.* study was that VPA strongly impaired the expression of HBZ [38]. The authors suggested that the opposite effect of VPA on Tax and HBZ expression might be caused by the nature of HDAC complexes present on the 5′- and 3′-HTLV-1 promoters in relation to their selective down-modulating properties. Alternatively, it cannot be excluded that activation of sense transcription by Tax and VPA might have impaired antisense transcription, either by competition for transcription factors or interference with its initiation. Indeed, Cavanagh *et al.* [10] have showed that, by deleting the 5′-LTR, sense transcription has a negative impact on antisense transcription (*i.e.*, from the 3′-LTR) [10]. Interestingly, despite increased Tax expression, Belrose *et al.* did not observe the expected increase in proviral load (PVL) in VPA-treated samples from HAM/TSP. Instead, a significant decrease of the PVL in VPA-treated samples from acute ATL patients was observed, suggesting a decrease in the percentage of ATL cells [120]. Using VPA to augment the level of histone acetylation and increase HTLV-1 gene expression in cultured cells from HAM/TSP patients, Mosley *et al.* [121] demonstrated that, while the level of Tax expression doubled after overnight treatment, the rate of CD8+ T-cell-mediated lysis of Tax-expressing cells was reduced by 50% [121]. VPA thus appeared to inhibit CD8+ T-cell-mediated cell from killing itself. These observations indicate that HDCAis may reduce the efficiency of CTL surveillance of HTLV-1. Further studies are needed to evaluate the use of HDIs in nonmalignant cases of HTLV-1 infection. Taken together, these observations strongly suggest that HBZ is a very interesting therapeutic target and that a therapy using VPA as part of the management of patients with acute and lymphoma ATL should be considered for the prevention of progression of chronic and smoldering ATL. Should a protective effect be shown, the long-standing safety profile of this compound would justify a prospective study in which its efficacy in preventing ATL in patients considered to be at high risk of disease is evaluated [122].

### 6.3. Immunotherapy

Another alternative approach is to target ATL cells using specific markers on the surface of the malignant cells with monoclonal antibodies. One of the first potential tested target is the interleukin (IL)-2alpha receptor α chain, CD25. Indeed, ATL cells express high level of CD25 on their surface. In their recent phase I/II trial on 34 patients with ATL, Berkowitz *et al.* [123] reported that daclizumab, a humanized monoclonal antibody which blocks IL-2 binding by recognizing CD25 on ATL cells, was associated with effective clinical responses in patients with indolent disease, although no beneficial responses were observed in patients with acute or lymphomatous subtypes of ATL [123]. The finding that daclizumab has antitumor activity and demonstrates a potential in achieving long-term responses in patients with the indolent form of ATL, suggest that immunotherapy offers a therapeutic option to prevent indolent diseases to develop into aggressive ATL [123].

Another target for immuno-based therapy against ATL is the CC chemokine receptor 4 (CCR4). CCR4 is principally expressed on regulatory T-cells (Tregs) and helper T-cells (Th), where it functions in inducing homing of these leukocytes to sites of inflammation. Tregs play an essential role in maintaining immune balance; however, in malignancy, Tregs impair host antitumor immunity and provide a favorable environment for tumors to grow [49]. Furthermore, ATL cells express high levels of CCR4 on their surface [124]. Mogamulizumab (KW-0761) is the first approved glyco-engineered therapeutic monoclonal antibody to target CCR4. 30% of patients with acute forms of ATL (5 out of 15) treated with Mogamulizumab showed a positive response [125]. Several ongoing clinical trials in Japan are investigating if combining Mogamulizumab with a chemotherapy treatment could be beneficial [126,127,128].

### 6.4. Stem Cell Transplantation

As a treatment strategy for ATL, allogeneic hematopoietic stem cell transplantation (allo-HSCT) with reduced intensity conditioning regimens (RIC) was prospectively evaluated. Several teams have reported the safety and feasibility of allo-HSCT with RIC using peripheral blood stem cells from an HLA-matched sibling donor in patients with acute ATL, who achieved remission after chemotherapy [129,130,131]. These studies showed that the overall survival (OS) at three years after allo-HSCT with RIC treatment ranged from 33% to 49% [132]. Interestingly, a significant decrease of the proviral load was also reported in many of these patients. These findings suggest that cell-mediated immunity to HTLV-1 was augmented in these patients, which might account for the efficacy of this therapy. In fact, Graft-*versus*-host disease (GVHD) is a good prognostic factor for ATL patients [130], indicating that an immune attack by donor lymphocytes is critical for the efficacy of treatment. Kanda *et al.* [133] reported that grade I/II acute GVHD was associated with a longer OS. Beneficial effects of allo-HSCT on non-Japanese ATL patients were recently confirmed by a retrospective study from the European Group for Blood and Marrow Transplantation's Lymphoma Working Party [134].

## 7. Perspectives and Conclusions

Although new therapeutic options are emerging, treatment of ATL patients remains challenging. The initial pathogenic event for ATL is HTLV-1 genomic integration; however, additional genetic alterations have also been implicated in ATL pathogenesis. Umino *et al.* [135] reported on the importance of clonal heterogeneity of ATL cells involving different genomic alterations; they further demonstrated that these cells originated from a common cell. It was suggested that approximately 70% of ATL cases undergo clonal evolution, and that genetic instability may contribute to the accumulation of genomic alterations [135]. In fact, the existence of multiple clones with genomic instability is one factor that renders ATL cells resistant to conventional chemotherapy. Even if a proportion of cells are killed by chemotherapy, new resistant clones likely emerge. Therefore, allo-HSCT might be efficient in curing ATL patients by eliminating HTLV-1-integrated recipient ATL clones through strong immune response, and subsequent replacement of the hematopoietic system with donor cells.

Whole genome sequencing revealed that carriers have 10^3^ to 10^4^ distinct clones with different HTLV-1 integration sites, and that most clones harbored one copy of HTLV-1 proviral DNA [136]. This indicates that HTLV-1 carriers potentially have 10^3^ to 10^4^ malignant clones. If the number of infected cells increases, there is a greater possibility that malignant transformation might occur. In order to reduce the number of pre-malignant cells in HTLV-1 carriers and thus prevent the development of ATL, treatment with HDAC inhibitors seems to be a promising strategy. Indeed vorinostat (suberoylanilide hygroxamic acid: SAHA), panobinostat (LBH-589) and MS-275 have been demonstrated to impede the growth of HTLV-1-infected cell lines and freshly isolated infected cells [137]. Furthermore, as reported by Belrose *et al.* [38], the link between VPA-induced apoptosis of HTLV-1-infected cell lines, decrease of proviral load in freshly isolated infected cells and loss of HBZ expression indicates that HBZ is a promising therapeutic target. However, further studies are needed to clarify the effect of HDACi on HBZ, although the inclusion of HDACi in clinical trials for the treatment of ATL is expected. Nevertheless, to increase the likehood of discovery of a cure for ATL, rigorous investigation remains necessary for optimizing therapeutic combinations, preventing ATL development in HTLV-1 carriers, and reducing the number of HTLV-1 carriers.
